# Effect of electroencephalogram-guided anesthesia on postoperative delirium in older adults after surgery: a systematic review and meta-analysis

**DOI:** 10.3389/fneur.2025.1638282

**Published:** 2025-08-28

**Authors:** Xiang Da, Xia Li, Anmin Dong, Li Chen, Youjia Ma, Xue Li

**Affiliations:** ^1^College of Health, Medicine and Wellbeing, The University of Newcastle, Newcastle, NSW, Australia; ^2^Department of Gastroenterology, The Second Affiliated Hospital of Chongqing Medical University, Chongqing, China

**Keywords:** postoperative delirium, older adults, meta-analysis, electroencephalogram-guided anesthesia, EEG

## Abstract

**Background:**

Postoperative delirium is a frequent and severe complication among elderly surgical patients. Electroencephalogram (EEG)-guided anesthesia, which optimizes sedation depth, holds promise for preventing postoperative delirium; however, current research findings remain inconsistent. This meta-analysis incorporates the most recent trials to evaluate the effectiveness of EEG-guided anesthesia in reducing postoperative delirium incidence in older adults.

**Methods:**

We conducted a comprehensive literature search of PubMed, the Cochrane Central Register of Controlled Trials, and Embase from their inception to February 1, 2025, to identify eligible studies. This systematic review and meta-analysis aimed to evaluate the effectiveness of electroencephalogram-guided anesthesia in preventing postoperative delirium among elderly surgical patients. Pooled effect estimates for all outcomes were calculated using a random-effects model. The quality and certainty of the evidence were assessed using the GRADE methodology. The primary outcome was the occurrence of postoperative delirium.

**Results:**

Of the 3,151 studies screened, 12 were deemed eligible for inclusion, encompassing a total of 7,441 patients, of whom 3,707 received EEG-guided anesthesia. Compared with standard care, EEG-guided anesthesia demonstrated a beneficial effect in reducing postoperative delirium among elderly patients (RR = 0.76, 95% CI: 0.61–0.96), as well as the incidence of postoperative infections (RR = 0.74, 95% CI: 0.58–0.95). Subgroup analyses revealed no significant interaction based on type of surgery (*p* = 0.18).

**Conclusion:**

EEG-guided anesthesia is associated with a reduced incidence of postoperative delirium in elderly surgical patients.

## Introduction

Postoperative delirium (POD) is a common and severe neurological complication in elderly surgical patients, with incidence rates ranging from 5 to 50% (1, 2). With the accelerating global aging population, it is projected that by 2050, the number of elderly individuals worldwide will reach 1.5 billion, which will significantly increase the public health burden associated with POD (3). POD has been shown to significantly prolong hospital stays and increase healthcare costs and is closely associated with postoperative cognitive decline and higher mortality rates (4). Therefore, identifying effective strategies for preventing POD has become a critical priority in perioperative care for older adults (5).

EEG-guided anesthesia involves real-time monitoring of brain electrical activity to precisely adjust anesthesia depth, preventing both excessive and insufficient anesthesia (6). Maintaining the bispectral index (BIS) within the range of 40–60 helps avoid burst suppression patterns, which are associated with postoperative cognitive dysfunction (6, 7). The real-time feedback from EEG monitoring enables anesthesiologists to fine-tune anesthetic dosages, reducing exposure to anesthesia and, consequently, lowering the risk of delirium (7). Furthermore, an optimal anesthetic depth helps stabilize intraoperative hemodynamics, preventing low perfusion and a decrease in cardiac output (8), which in turn reduces immune suppression (9, 10), minimizes intraoperative stress (10), and alleviates inflammatory responses, thereby decreasing the risk of postoperative infections (11). In particular, in elderly patients with weakened immune systems, EEG-guided anesthesia may enhance hemodynamic stability and immune function, providing additional protection against postoperative infections (8–10).

A previous meta-analysis (12) combining five randomized controlled trials with a total of 3,433 participants found no significant association between EEG-guided anesthesia and a reduced incidence of POD. Trial sequential analysis indicated that the required sample size was 4,180 participants, suggesting insufficient sample size and limiting the robustness of the conclusions. Recent studies (13–15) have reported inconsistent findings, further highlighting the ongoing debate in this field. To address this, we have incorporated four of the most recent randomized controlled trials in our analysis to provide a more comprehensive comparison and further validate the potential of EEG-guided anesthesia in reducing POD and postoperative infections.

We conducted a systematic review and meta-analysis, including only randomized controlled trials evaluating the effects of EEG-guided anesthesia on postoperative outcomes in elderly patients. This study aims to provide more precise evidence to inform clinical guidelines and enhance perioperative neuroprotection and comprehensive management strategies for the elderly.

## Methods

### Protocol and guidance

This study was conducted in strict accordance with the Preferred Reporting Items for Systematic Reviews and Meta-Analyses (PRISMA) guidelines (16), and the research protocol was registered in the International Prospective Register of Systematic Reviews (PROSPERO, https://www.crd.york.ac.uk/PROSPERO) under registration number CRD420251053668.

### Information sources and search strategy

This study conducted a systematic search of Cochrane Central Register of Controlled Trials (CENTRAL), PubMed, MEDLINE, Web of Science, Embase, and ClinicalTrials.gov. The search spanned from the inception of each database to February 1, 2025, encompassing preprints and articles published online ahead of print. To supplement the search, reference lists of included studies and relevant systematic reviews were examined, conference abstracts and grey literature (including unpublished reports) were screened, and experts in the field were consulted. No restrictions on language, country, or region were applied to minimize language and geographical bias. Literature screening was independently performed by two reviewers (XD and LC) in a blinded manner, with discrepancies resolved through discussion and adjudication by a third reviewer (XL). Studies were eligible for inclusion if their titles, abstracts, original titles, substance names, subject headings, keyword fields, or unique identifiers contained all of the following Medical Subject Headings (MeSH) and keywords: “Electroencephalography,” “postoperative delirium,” “elderly patients,” “older patients,” “aged patients,” “geriatric patients,” and “randomized controlled trial.”

### Eligibility criteria

The studies included in this paper were selected according to the PICOS criteria, as outlined below:

*Population*: this meta-analysis included studies on adults with an average age of over 60 years who underwent different surgeries under general anesthesia.

*Intervention*: included studies evaluated EEG-guided anesthesia management, using real-time brain activity monitoring (e.g., bispectral index or spectral edge frequency) to guide anesthesia protocols.

*Comparison intervention*: control groups received standard anesthesia care without EEG monitoring, with anesthesia depth adjusted by clinical signs (heart rate, blood pressure) or physician experience.

*Outcome*: the primary outcome was postoperative delirium. Secondary outcomes included length of hospital stay, postoperative infection and all-cause mortality at the longest follow-up.

*Study design*: only randomized controlled trials were included.

### Study selection

Two authors, XD and LC, independently undertook the screening of titles and abstracts obtained through systematic search. After the initial screening, the same two authors conducted full-text reviews of the selected articles. During this process, any discrepancies were resolved through discussion between the two to maximize the reduction of bias. In cases where consensus could not be reached, a reviewer would make the final decision.

### Data extraction

Data were independently extracted by two reviewers (XD and LC) using a predesigned data extraction form. Extracted variables included author, year, country, study design, sample size, mean or median age, sex distribution, type of surgery (cardiac vs. non-cardiac), EEG monitoring modality (e.g., BIS, raw EEG), method and timing of delirium assessment (e.g., CAM, CAM-ICU, DSM-IV), postoperative infection definition, and duration of follow-up for mortality. Long-term outcomes were included when reported. All extracted data were cross-checked by a third reviewer (XL).

### Risk of bias assessment

Two independent reviewers, XD and LC, assessed the risk of bias in the included studies based on the guidelines outlined in the Cochrane Risk of Bias Assessment Tool (17). This tool evaluates bias across seven domains—participant involvement, attrition, measurement of prognostic factors, outcome assessment, confounding variables, and statistical analysis and reporting—providing a comprehensive appraisal of methodological quality. Studies were categorized as having a high risk of bias if they were rated as “high” in one or more domains. A low risk of bias was assigned when all six domains were rated as “low.” Studies not meeting the criteria for either low or high risk were classified as having a moderate risk of bias. The systematic approach ensures a thorough evaluation of potential sources of bias within the included trials. Disagreements between the two reviewers were resolved through detailed discussion. If consensus could not be reached, a third reviewer (XL) made the final decision.

### Confidence of evidence

Two independent authors, XD and LC, conducted a systematic evaluation using the Grading of Recommendations Assessment, Development and Evaluation (GRADE) approach (18). This methodology comprehensively assesses trials across dimensions such as study design, risk of bias, inconsistency, imprecision, and indirectness. Subsequently, evidence was classified into four grades—high, moderate, low, or very low—based on the aforementioned criteria. In the event of discrepancies between reviewers, discussions were prioritized for resolution; if consensus remained unattained, a reviewer would make the final ruling.

### Data analysis

Statistical analyses were conducted following a predefined meta-analysis protocol using Review Manager software (version 5.4.1, Cochrane Collaboration). Given the clinical and methodological variability among included studies, a random-effects model was applied to each study’s data to account for between-study heterogeneity, providing more conservative and generalizable pooled effect estimates.

For dichotomous outcomes, relative risks with corresponding 95% confidence intervals were calculated, while mean differences were used for continuous outcomes. Two-sided *p*-values less than 0.05 were considered statistically significant (19). To assess methodological rigor and the consistency of results, heterogeneity was quantified using the I^2^ statistic, with thresholds defined as follows: I^2^ < 25% indicating low heterogeneity, 25–50% moderate, 50–75% substantial, and >75% considerable heterogeneity (20, 21). Publication bias was evaluated through visual inspection of funnel plots to identify potential small-study effects influencing the overall findings.

### Sensitivity analysis

To assess the robustness of our findings, we performed a sensitivity analysis using the following approaches: (1) using a fixed-effect model, (2) excluding trial assessed as unclear or high-risk, and (3) each study was systematically excluded in turn.

### Subgroup analysis

To better understand the potential sources of heterogeneity in the effectiveness of EEG-guided anesthesia for preventing postoperative delirium in elderly patients, subgroup analyses of the primary outcome were conducted based on the following criteria: type of surgery (Cardiac surgery vs. Non-cardiac surgery).

## Results

**Figure 1:**
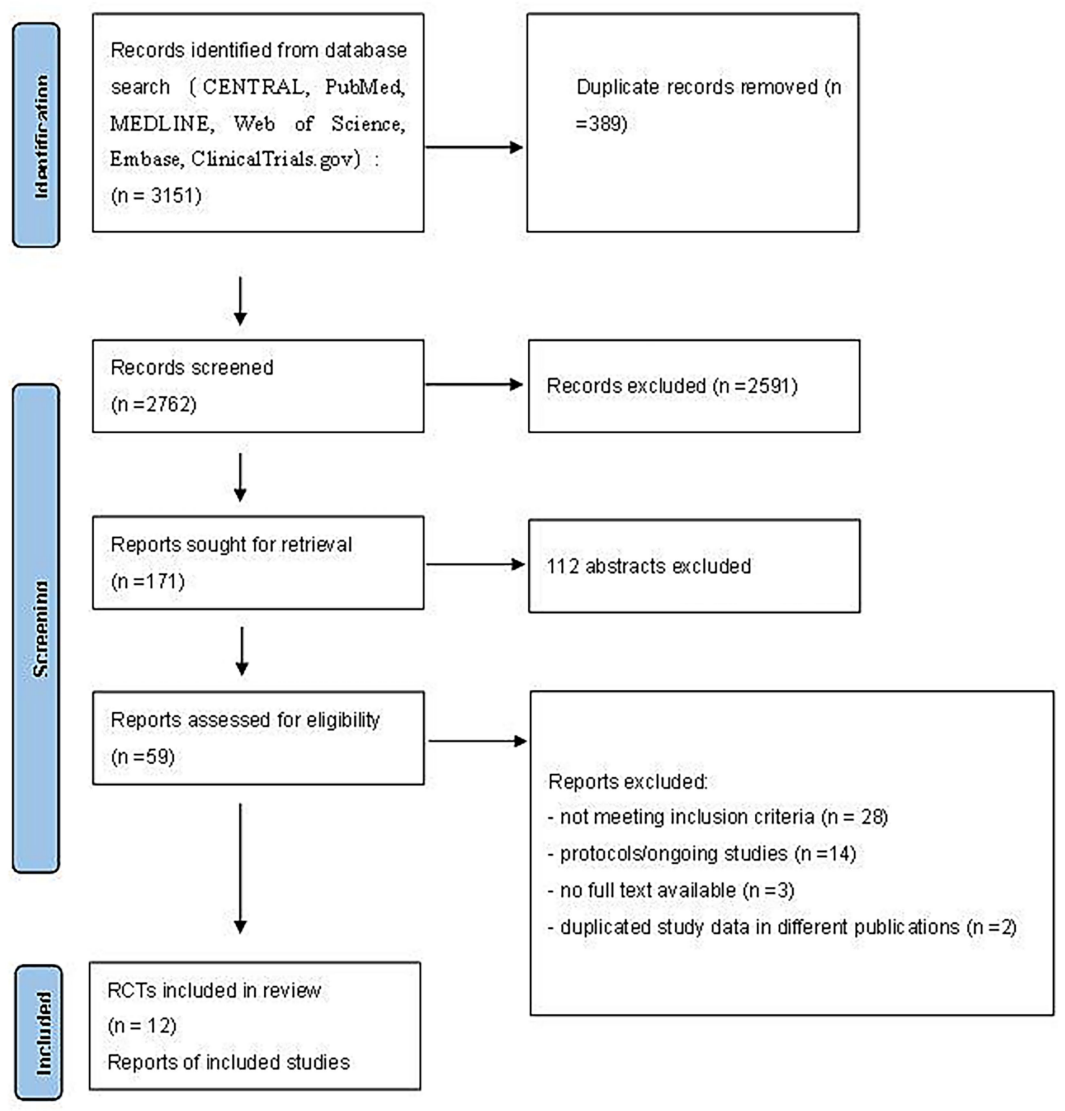
Search strategy and final included and excluded studies.

Our initial literature search identified 3,151 records. After removing duplicates, 2,762 unique articles remained for screening. Following a rigorous assessment of titles, abstracts, and full texts, 12 randomized controlled trials were deemed eligible for inclusion in this systematic review (Figure 1). Table 1 summarizes the characteristics of each trial included in this analysis. These trials were published between 2013 and 2024, with sample sizes ranging from 81 to 1,545 patients. The included studies exhibited a certain degree of heterogeneity in terms of participant characteristics and study protocols. Although the average age of participants was over 60 years, variations were observed in sex distribution and types of surgery. In addition, intraoperative electroencephalogram (EEG) monitoring methods varied across studies. Eight trials (14, 22–27) employed the bispectral index (BIS) for EEG monitoring, while three studies (13, 28, 29) utilized the SEDLine Brain Function Monitor to assess brain activity. The tools used to evaluate postoperative delirium also differed: eight studies (13, 15, 22, 24–26, 28, 29) used the Confusion Assessment Method (CAM), one study (27) applied both the 3D Confusion Assessment Method (3D-CAM) and the Intensive Care Unit version of CAM (CAM-ICU), and another study (23) adopted the Diagnostic and Statistical Manual of Mental Disorders, Fourth Edition (DSM-IV) criteria. The timing of delirium assessments ranged from once daily until postoperative day 3 or 7. Furthermore, several studies reported long-term outcomes: three (13, 14, 30) evaluated 30-day mortality, two (22, 23) assessed 90-day mortality, and another three (15, 25, 27) reported 1-year mortality. The remaining studies did not report relevant data.

**Table 1:** Characteristics of the included RCTs.

First authors	Year	Number of participants	Sex distribution		Age, mean ± SD (years)		Countries	Type of pEEG monitor	Delirium assessment method	Mortality follow-up period	Type of surgery
			EEG Female, *n* (%)	Usual care Female, *n* (%)	EEG	Usual care					
Chan et al.	2013	902	37.8	39.6	68.1 ± 8.2	67.6 ± 8.3	China	BIS	CAM	3 months	Elective major surgery expected to last 2 h
Radtke et al.	2013	1,155	44.7	47.6	69.7 ± 6.3	70.1 ± 6.5	Germany	Bilateral BIS	DSM IV criteria applied by psychiatrists	3 months	Elective surgery expected to last 60 min or more
Zhou et al.	2018	81	29.3	32.5	68.29 ± 2.81	68.90 ± 2.99	China	BIS	CAM	NR	Colorectal surgery with expected duration >2 h
Wildes et al.	2019	1,213	45.9	45.5	69.7 ± 2.4	69.8 ± 2.8	USA	BIS	CAM or CAM-ICU and supplemental note review	30 days	Major elective surgery excluding neurosurgery
Kunst et al.	2020	82	21.4	15.0	71.6 ± 5.0	72.0 ± 4.3	UK	BIS	CAM	1 year	Cardiac surgery
Tang et al.	2020	204	52.0	52.0	72.1 ± 5.6	71.8 ± 5.3	USA	SEDLine brain function monitor	CAM	NR	Major elective noncardiac surgery, excluding neurosurgery
Brown et al.	2021	217	61.8	67.0	72.5 ± 2.0	72.3 ± 1.8	USA	BIS	CAM	NR	Noncardiac surgery
Evered et al.	2021	515	36.0	35.1	70.8 ± 6.9	71.1 ± 6.8	Australia, China, USA	BIS	3D-CAM or CAM-ICU	1 year	Major noncardiac surgery with expected duration >2 h
Xu et al.	2021	255	14.2	11.7	62.31 ± 8.02	63.16 ± 7.17	China	SEDLine brain function monitor	CAM	NR	Carotid Endarterectomy
Wang et al.	2022	1,545	43.5	40.7	61.5 ± 3.0	61.0 ± 3.0	China	SEDLine brain function monitor	CAM	30 days	Laparoscopic Surgery
He et al.	2023	141	36.1	32.8	68.7 ± 5.9	69.3 ± 6.7	China	BIS	Narcotrend	30 days	elective major abdominal surgery
Deschamps et al.	2024	1,131	21.9	27.6	70.0 ± 2.5	71.3 ± 2.3	Canada	NR	CAM	1 year	cardiac surgery

pEEG, processed electroencephalography; CAM, Confusion Assessment Method; CAM-ICU, Confusion Assessment Method for the Intensive Care Unit; DSM-IV, Diagnostic and Statistical Manual of Mental Disorders, Fourth Edition; 3D-CAM, 3-Minute Diagnostic Interview for CAM-Defined Delirium; NR, not reported; UK, United Kingdom; US, United States.

**Figure 2:**
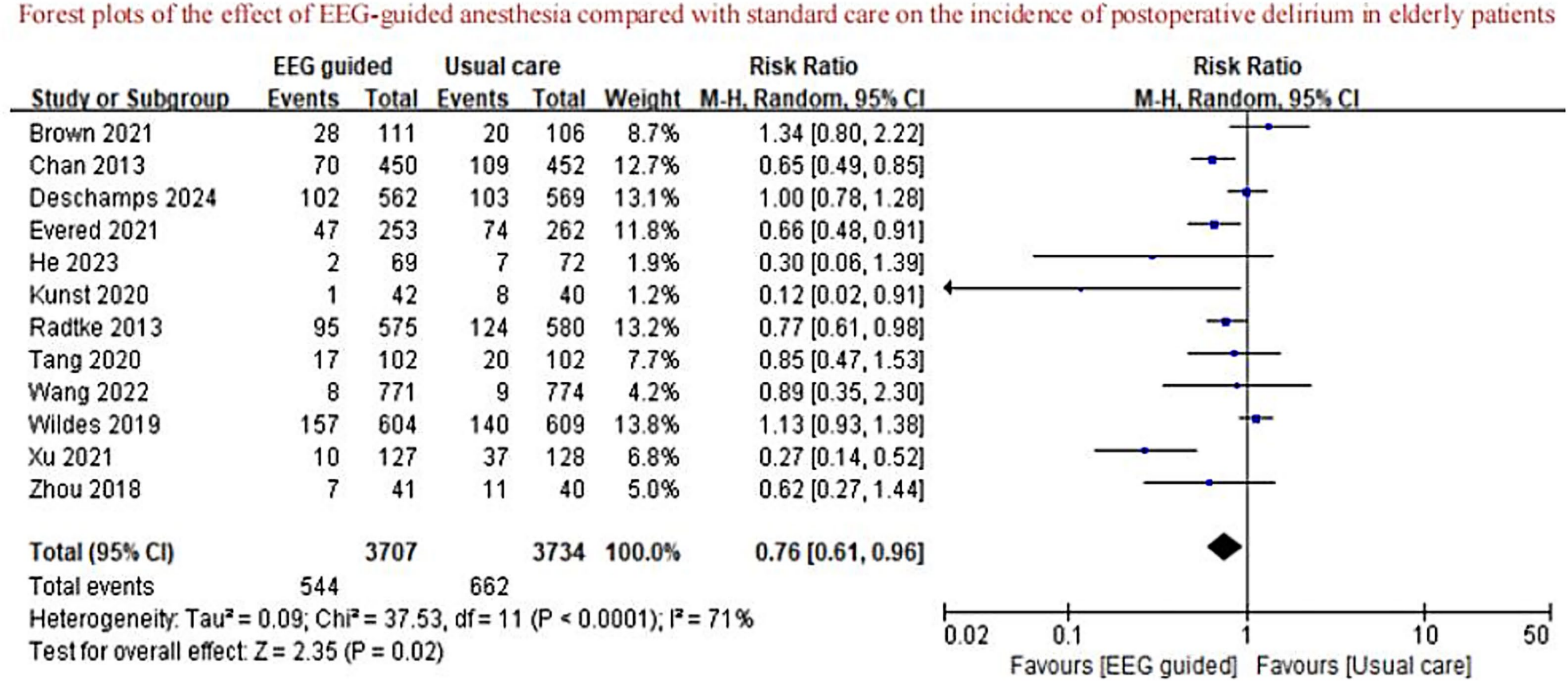
Forest plots of the effect of EEG-guided anesthesia compared with standard care on the incidence of postoperative delirium in elderly patients.

**Figure 3:**
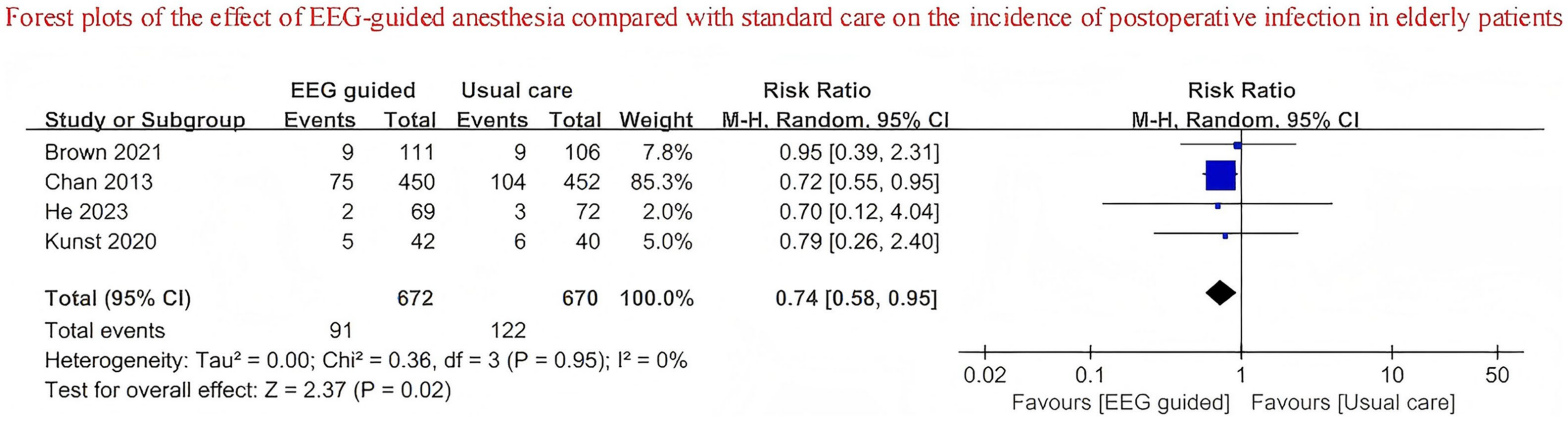
Forest plots of the effect of EEG-guided anesthesia compared with standard care on the incidence of postoperative infection in elderly patients.

**Figure 4:**
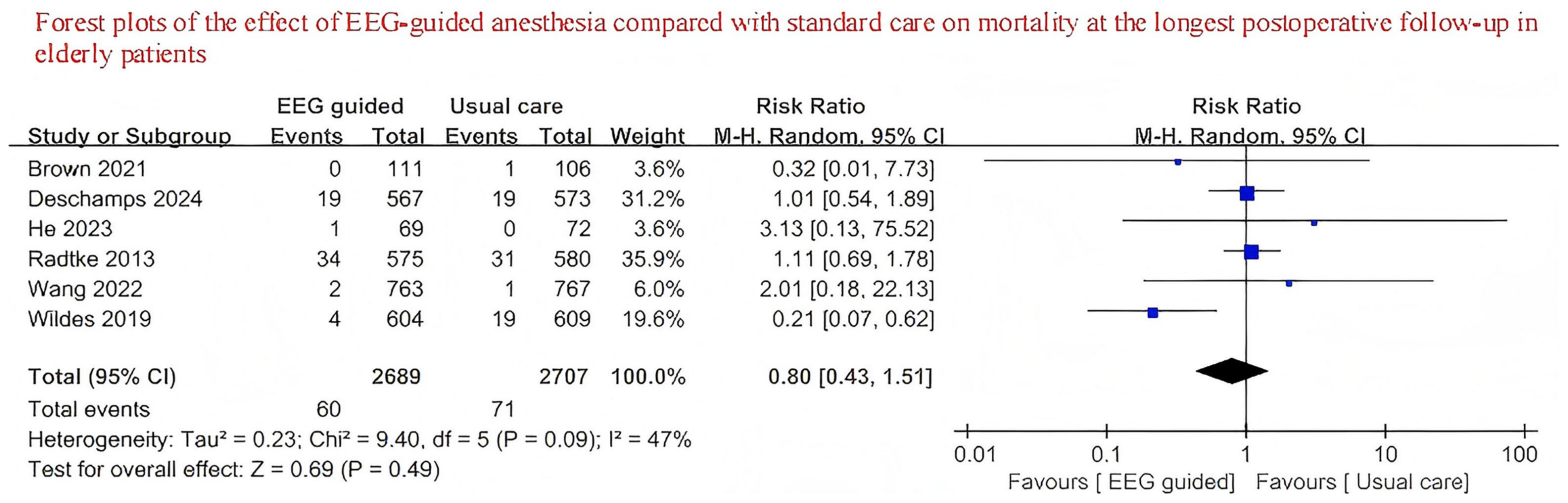
Forest plots of the effect of EEG-guided anesthesia compared with standard care on mortality at the longest postoperative follow-up in elderly patients.

**Figure 5:**
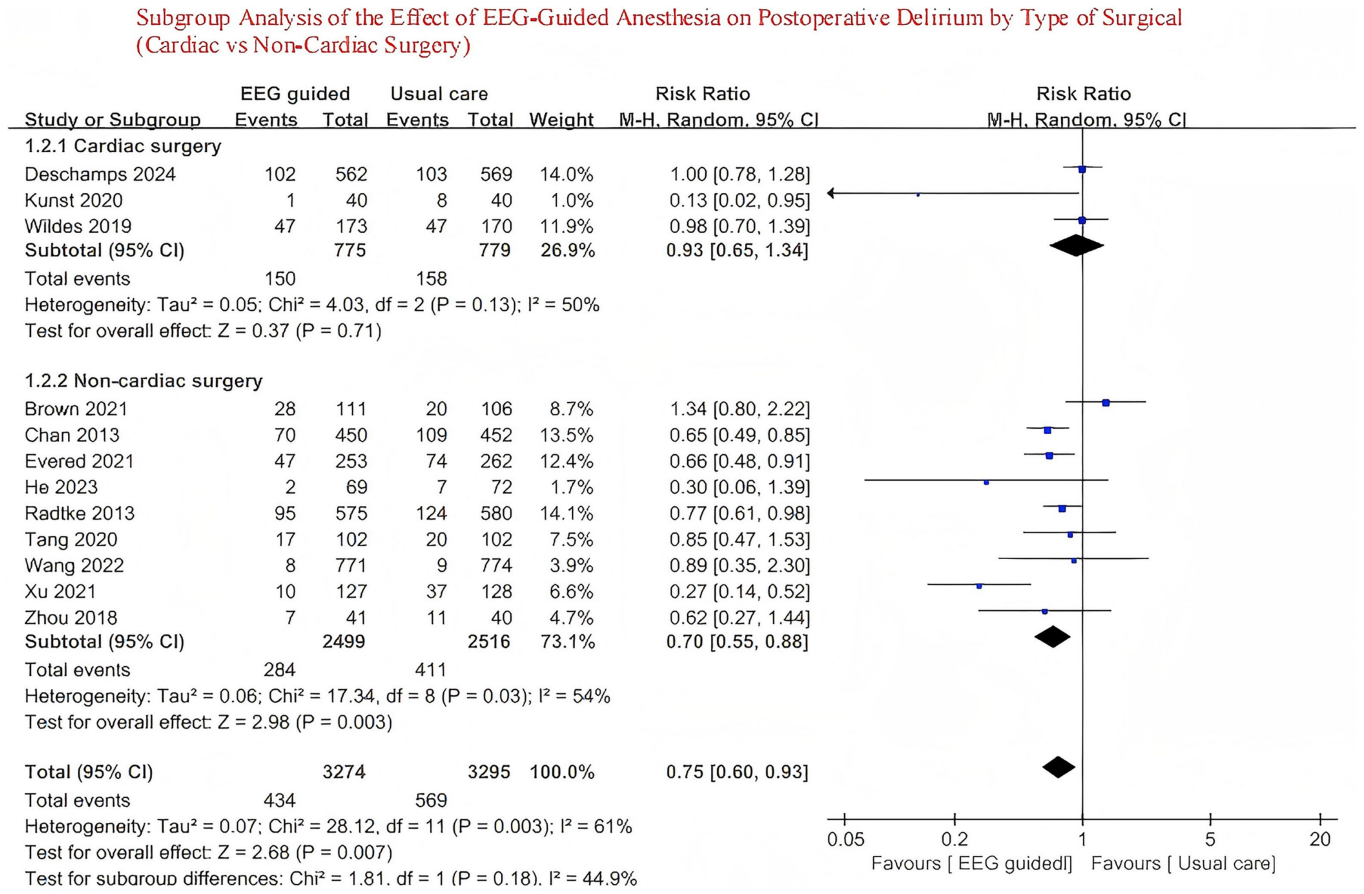
Subgroup analysis of the effect of EEG-guided anesthesia on postoperative delirium by type of surgical (cardiac vs. non-cardiac surgery).

A total of 3,707 patients received EEG-guided anesthesia, with a lower incidence of postoperative delirium observed compared to 3,734 patients in the standard care group. The overall incidence of postoperative delirium was 14.94% (554/3,707) in the EEG-guided group and 17.73% (662/3,734) in the standard care group. The pooled relative risk for postoperative delirium was 0.76 (95% CI: 0.61–0.96) in the EEG-guided group compared to the standard care group (Figure 2). In addition, the incidence of postoperative infections was lower in the EEG-guided group, with an incidence of 13.54% (91/672) versus 18.21% (122/670) in the standard care group (RR = 0.74; 95% CI: 0.58–0.95) (Figure 3). However, the intervention demonstrated no significant effect on all-cause mortality at the longest follow-up among elderly patients. The mortality incidence was 2.23% (60/2,689) in the EEG-guided group and 2.62% (71/2,707) in the standard care group (RR = 0.80; 95% CI: 0.43–1.51) (Figure 4). Subgroup analyses revealed no statistically significant interaction based on type of surgery (*p* for interaction = 0.18) (Figure 5). We performed a sensitivity analysis using a fixed-effects model, excluding studies with unclear or high risk of bias. Additionally, each study was sequentially removed to assess its impact on the overall results. The findings remained robust throughout all analysis(Supplementary Table 2). The funnel plot for the primary outcome appeared symmetrical, suggesting no significant publication bias (Supplementary Figure 3).

Table 1 in the Data Supplement summarizes the risk-of-bias assessment. Among the included trials, six were rated as having a low risk of bias. Four studies were judged to have some unclear, primarily due to potential biases related to intervention implementation and outcome reporting. The remaining two trials were assessed as high risk, mainly attributable to deficiencies in outcome measurement and selective reporting. Based on the GRADE evaluation, the overall certainty of the evidence for the primary outcome was deemed low.

## Discussion

This meta-analysis included 12 randomized controlled trials with a total of 7,441 elderly surgical patients, among whom 3,707 received EEG-guided anesthesia. Our findings demonstrate that EEG-guided anesthesia significantly reduces the incidence of postoperative delirium (RR = 0.76; 95% CI: 0.61–0.96) and postoperative infection (RR = 0.74; 95% CI: 0.58–0.95), while showing no statistically significant effect on long-term all-cause mortality (RR = 0.80; 95% CI: 0.43–1.51). These findings strengthen the evidence supporting the role of EEG guidance in perioperative neuroprotection and are consistent with our predefined hypothesis (Table 2).

**Table 2:** Summary of findings and strength of evidence.

Outcome	No. of patients (trials)	Relative effect, risk ratio (95% CI)	Absolute effect estimates (per 1,000)			Quality of the evidence
			EEG-guided	Usual care	Difference	
The primary outcome						
Postoperative delirium	7,441 (12)	0.85 (0.73, 0.98)	133	173	−43 [−12, −69]	Moderate^*^
The secondary outcome						
Postoperative infection	1,342 (4)	0.74 (0.58, 0.95)	135	182	−47 [−9, −76]	High
Mortality at the longest postoperative follow-up	5,396 (6)	0.80 (0.43, 1.51)	22	26	−5 [−15, 13]	High

* Inconsistency.

CI, confidence interval; RR, risk ratio.

This meta-analysis, encompassing 7,441 participants from 12 trials (13–15, 22–29), demonstrates that EEG-guided anesthesia is effective in reducing the incidence of postoperative delirium in elderly patients. Our findings differ from previous meta-analysis (12), which did not observe a significant reduction in the incidence of postoperative delirium among elderly patients receiving EEG-guided anesthesia. However, that study acknowledged the limitation of an insufficient sample size. The trial sequential analysis conducted in the previous review estimated that a minimum sample size of 4,180 participants was required to achieve adequate statistical power. However, with only 3,433 participants included, the study may have been underpowered, raising the possibility of a type II error. In addition, several randomized controlled trials (13–15) published in recent years have reported inconsistent findings, underscoring the ongoing uncertainty in this field. So, our meta-analysis incorporates a larger and more up-to-date collection of randomized controlled trials (13–15, 22–29), including a total of 7,441 participants, substantially improving the statistical power and reliability of the conclusions. The results indicate that EEG-guided anesthesia is associated with a reduced risk of Postoperative Delirium in older patients. This finding not only enriches the existing evidence base but also suggests that the potential benefits of Electroencephalogram monitoring in preventing Postoperative Delirium are becoming increasingly apparent as more data accumulates. We also performed subgroup analyses based on surgical sites, which revealed no significant difference between the subgroups.

Our results regarding postoperative infection are in agreement with previous study (8), which suggests that optimal anesthetic depth can stabilize intraoperative hemodynamics and reduce systemic inflammatory responses, thereby lowering the risk of infection. EEG monitoring enables individualized anesthetic titration, helping to prevent hypotension (31), hypoperfusion (32), and overexposure to anesthetic agents (33)—all of which have been associated with postoperative immune suppression. In elderly patients, who are more vulnerable to infection due to immunosenescence (34), EEG-guided anesthesia may offer additional protection by preserving physiological stability and reducing surgical stress (30).

In contrast, our findings did not demonstrate a statistically significant reduction in long-term all-cause mortality. This result differs from several prior studies (35, 36) that have reported potential survival benefits associated with optimized anesthesia depth. One possible explanation is the limited number of trials (*n* = 4) and patients contributing to this specific outcome, resulting in wide confidence intervals and reduced statistical power. Therefore, this may represent a type II error or false-negative finding. Further large-scale trials are warranted to validate this association.

The mechanisms by which EEG-guided anesthesia may reduce the risk of postoperative delirium remain incompletely understood. One proposed explanation is that maintaining the bispectral index within the range of 40–60 ^(6,7)^ during surgery helps to avoid excessively deep anesthesia and reduces the occurrence of burst suppression patterns, which have been strongly associated with postoperative cognitive impairment. EEG monitoring provides real-time feedback on cerebral activity, enabling anesthesiologists to more precisely titrate anesthetic dosages and thereby potentially lower the risk of delirium (37). Studies (38, 39) have shown that both inhalational and intravenous anesthetics may induce neuronal and glial cell damage or apoptosis when used in high doses or for prolonged periods—particularly in brain regions such as the hippocampus, which are closely related to cognitive function. EEG-guided anesthesia may help minimize unnecessary exposure to anesthetics, thereby preserving neural integrity and further reducing the likelihood of Postoperative Delirium (37).

This meta-analysis indicates that EEG-guided anesthesia significantly reduces the incidence of postoperative delirium in elderly patients. Given the substantial impact of POD on recovery and cognitive outcomes, EEG monitoring may serve as a practical strategy to enhance anesthesia safety in this population. However, the underlying mechanisms remain unclear (40). Future research should explore the relationship between anesthetic depth, burst suppression, and cognitive outcomes, and conduct large-scale, multicenter randomized controlled trials to further assess its efficacy and clinical feasibility.

This study has several limitations. First, there was considerable heterogeneity in the diagnostic criteria, assessment tools (e.g., CAM-ICU, 3D-CAM), and follow-up durations used across the included studies. Second, variations in anesthetic agents, dosages, and delivery methods (including inhalational VS. intravenous anesthesia) across trials may have influenced the overall results.

## Conclusion

Our study suggests that EEG-guided anesthesia may contribute to a lower incidence of postoperative delirium in elderly surgical patients, highlighting its potential as an effective strategy in perioperative neuroprotection.

## Data Availability

The datasets presented in this study can be found in online repositories. The names of the repository/repositories and accession number(s) can be found in the article/Supplementary material.
