# Supplementary material for: Effect of electroencephalogram-guided anesthesia on postoperative delirium in older adults after surgery: a systematic review and meta-analysis
Source: Front Neurol. 2025 Aug 28;16:1638282. doi: 10.3389/fneur.2025.1638282 (PMC12422923; doi:10.3389/fneur.2025.1638282)

**Appendix**

Figure 1: Risk of bias summary

Table 2: Sensitivity analysis of meta-analysis

Table 3: Funnel Plot Assessing Publication Bias in the Meta-Analysis of EEG-Guided Anesthesia and POD

Figure 1: Risk of bias summary


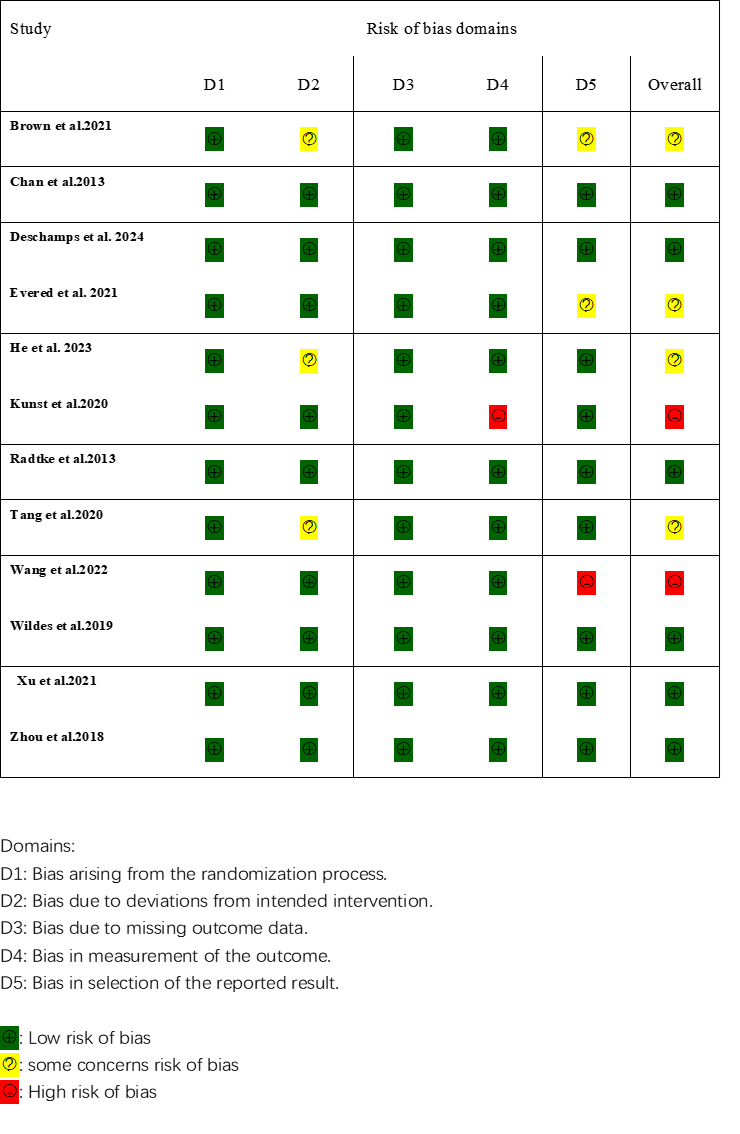


Table 2: Sensitivity analysis of meta-analysis

|  | NO. patients(trials) | RR | 95%CI |
| --- | --- | --- | --- |
| All trials | 7441(12) | 0.76 | 0.61,0.96 |
| Using fixed-effect models | 7441(12) | 0.83 | 0.75,0.92 |
| Excluding studies with high or unknown risk of bias | 4737（6） | 0.85 | 0.76,0.95 |
| Excluding each randomized control trial in turn |  |  |  |
| Excluding Brown et al.2021 | 7224(11) | 0.81 | 0.73,0.90 |
| Excluding Chan et al.2013 | 6539(11) | 0.86 | 0.77,0.96 |
| Excluding Deschamps et al. 2024 | 6310(11) | 0.80 | 0.71,0.89 |
| Excluding Evered et al. 2021 | 6926(11) | 0.85 | 0.76,0.95 |
| Excluding He et al. 2023 | 7300(11) | 0.83 | 0.75,0.92 |
| Excluding Kunst et al.2020 | 7359(11) | 0.84 | 0.76,0.93 |
| Excluding Radtke et al.2013 | 6286(11) | 0.84 | 0.75,0.94 |
| Excluding Tang et al.2020 | 7237(11) | 0.83 | 0.75,0.92 |
| Excluding Wang et al.2022 | 5896(11) | 0.83 | 0.75,0.92 |
| Excluding Wildes et al.2019 | 6228(11) | 0.75 | 0.66,0.84 |
| Excluding Xu et al.2021 | 7186(11) | 0.86 | 0.78,0.95 |
| Excluding Zhou et al.2018 | 7360(11) | 0.83 | 0.75,0.92 |

Table 3．Funnel Plot Assessing Publication Bias in the Meta-Analysis of EEG-Guided Anesthesia and POD


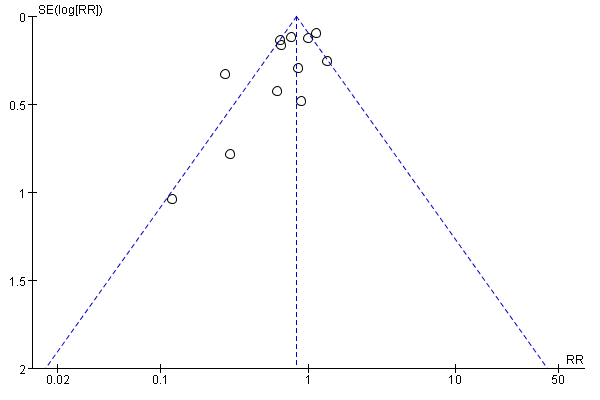

Supplement: Supplementary file 1 [file Table_1.docx]
